# Young Patients with Suspected Uncomplicated Renal Colic are Unlikely to Have Dangerous Alternative Diagnoses or Need Emergent Intervention

**DOI:** 10.5811/westjem.2015.1.23272

**Published:** 2015-03-13

**Authors:** Elizabeth M. Schoenfeld, Kye E. Poronsky, Tala R. Elia, Gavin R. Budhram, Jane L. Garb, Timothy J. Mader

**Affiliations:** * Baystate Medical Center/Tufts School of Medicine, Department of Emergency Medicine, Boston, Massachusetts; † Baystate Medical Center/Tufts School of Medicine, Epidemiology/Biostatistics, Department of Academic Affairs, Boston, Massachusetts

## Abstract

**Introduction:**

In the United States there is debate regarding the appropriate first test for new-onset renal colic, with non-contrast helical computed tomography (CT) receiving the highest ratings from both Agency for Healthcare Research and Quality and the American Urological Association. This is based not only on its accuracy for the diagnosis of renal colic, but also its ability to diagnose other surgical emergencies, which have been thought to occur in 10–15% of patients with suspected renal colic, based on previous studies. In younger patients, it may be reasonable to attempt to avoid immediate CT if concern for dangerous alternative diagnosis is low, based on the risks of radiation from CTs, and particularly in light of evidence that patients with renal colic have a very high likelihood of having multiple CTs in their lifetimes. The objective is to determine the proportion of patients with a dangerous alternative diagnosis in adult patients age 50 and under presenting with uncomplicated (non-infected) suspected renal colic, and also to determine what proportion of these patients undergo emergent urologic intervention.

**Methods:**

Retrospective chart review of 12 months of patients age 18–50 presenting with “flank pain,” excluding patients with end stage renal disease, urinary tract infection, pregnancy and trauma. Dangerous alternative diagnosis was determined by CT.

**Results:**

Two hundred and ninety-one patients met inclusion criteria. One hundred and fifteen patients had renal protocol CTs, and zero alternative emergent or urgent diagnoses were identified (one-sided 95% CI [0–2.7%]). Of the 291 encounters, there were 7 urologic procedures performed upon first admission (2.4%, 95% CI [1.0–4.9%]). The prevalence of kidney stone by final diagnosis was 58.8%.

**Conclusion:**

This small sample suggests that in younger patients with uncomplicated renal colic, the benefit of immediate CT for suspected renal colic should be questioned. Further studies are needed to determine which patients benefit from immediate CT for suspected renal colic, and which patients could undergo alternate imaging such as ultrasound.

## INTRODUCTION

Currently there are several guidelines suggesting that the standard of care for the diagnosis of new-onset acute renal colic is a non-contrast helical Computed Tomography (CT).^1^,^2^ Due to the accuracy of CT, its use in suspected renal colic has jumped from 4% to 42.5% from 1996–2007, resulting in over 500,000 CTs performed for renal colic every year in the United States.^3^,^4^ While multiple studies have suggested that CTs for first time renal colic should be routine because of the risk of dangerous alternative diagnoses, there is no evidence that increased utilization has changed outcomes or even increased the rate of dangerous alternative diagnoses detected.^1^–^3^,^5^–^8^ Additionally, the risks of radiation have become increasingly apparent to both the medical community and to our patients, and only a minority of patients with uncomplicated renal colic will eventually require urological intervention, with a smaller minority requiring an emergent intervention. 9–14 While clinicians appreciate both the confirmation of stone presence and being able to prognosticate based on the results of the CT, as stone location and size do influence spontaneous passage rates, immediate CT does not change management in the majority of cases.^14^,^15^ Furthermore, it is well documented that patients with renal colic are at risk for multiple CTs during their lifetimes.^16^

Recent studies have attempted to help clinicians predict which patients will have kidney stones prior to CT, with the added benefit that the higher the likelihood of stone, the lower the likelihood of a dangerous alternative diagnosis.^17^ Moore et al.^18^ demonstrated that the rate of dangerous alternative diagnoses of 10% demonstrated in older retrospective studies was likely an overestimate based on research methodology.^5^ While at least one study noted that the only dangerous alternative diagnoses found were in older patients, no studies have attempted to use age to risk stratify patients with suspected renal colic and their risk of dangerous alternative diagnoses.^16^

A recent, large, multi-centered comparative effectiveness trial suggested that “ultrasound-first” is a reasonable approach to renal colic in the ED.^19^ However, 27–40% of patients who received “ultrasound-first” went on to have a CT during their visit. While comprehensive, this study did not help distinguish between those who need a CT for identification of a dangerous alternative diagnosis or for the planning of a urological intervention from those who do not see a change in management based on that CT. Although not yet directly studied, we suspect that age may play a role in this question.

We hypothesized that in non-pregnant adult patients age 50 and under who present with flank pain but without pyuria or trauma, the incidence of dangerous alternative diagnoses would be low (less than 3%,) and the rate of immediate urologic intervention would also be low (less than 5%).

## METHODS

### Study Design

This study was approved by the local institutional review committee for human subjects. This was a retrospective chart review of all non-pregnant patients age 18–50 years presenting to an urban, tertiary care Emergency Department (ED) with the chief complaint of “flank pain” or “suspected kidney stone” during a 12-month period in 2011–2012. Electronic medical records were used so all identified visits had usable, legible records. Attention was paid to previous criticisms of retrospective chart reviews in order to decrease bias and maintain methodologic quality.^20^–^22^ Specifically, abstractors were trained, inclusion and exclusion criteria were clearly delineated *a priori*, data abstraction forms were used with defined variables, abstractors’ performance was monitored and compared via interrater reliability testing, the sampling method was determined *a priori*, and the study was IRB approved.^22^ We were unable, due to staffing, to make data abstractors blind to the hypothesis of the study, and because we found little missing data of importance as we piloted our chart review, we did not have a systematic plan for dealing with missing data. Regarding more recent criticisms of chart review methodology,^21^ we did perform sample size calculations *a priori*, we have included the data collection form (Appendix), we have included a flow diagram regarding inclusion and exclusion (Figure), we piloted the chart review process and avoided decrement of accuracy of coding by having frequent meetings to discuss questions, and we chose to measure interrater reliability for questions that were most likely to affect the outcome of the study (Table 4).

### Study Setting and Population

This ED has a combined pediatric and adult volume of >110,000 visits per year. The ED is staffed by attending physicians, residents and physician assistants, and CT is available 24 hours a day, 7 days a week, with attending radiologist interpretation available until midnight and resident preliminary interpretations for 8 hours overnight.

### Protocol

Electronic medical records were queried for coded triage complaints of “flank pain” or “possible kidney stone” from June 2011 until May of 2012. Patients were included if they complained of flank pain, were 18–50 years old, and did not have any exclusion criteria. Diagnoses were available as part of original query and could be viewed prior to chart review allowing exclusion of patients with the following diagnoses: trauma, pregnancy, or urinary tract infection (UTI)/pyelonephritis. Remaining charts were individually reviewed by the authors based on a standardized data collection form and an *a priori* coding plan and charts were then excluded based on the inclusion/exclusion criteria (Figure). Patients were included regardless of whether or not they had a history of kidney stones. Exclusion criteria included: left without being seen, no physician note, painless hematuria, UTI/pyelonephritis, trauma causing chief complaint (major or minor), pregnancy known or discovered during visit, end stage renal disease on hemodialysis/peritoneal dialysis or kidney transplant, recent surgical or urological intervention (60 days), already seen for this episode of pain (and index visit captured), or no “flank pain” in physician note. If the patient had a visit within the past 60 days for the same complaint, we checked to see if it was captured by the original query, and if not, the first of the two visits was used, provided it met inclusion criteria. If a previous visit for a similar complaint occurred more than 60 days before captured visit, it was considered another discrete episode and could be included.

All reviewers were trained in the use of the standardized data collection form by the PI, and after training each sub-investigator and research assistant collected data on at least 10 charts with the PI present. Additionally, 10% of the charts were assessed by the PI and another data abstractor for measurements of inter-rater reliability. Data was entered into a RedCap database. The database was reviewed for inconsistencies (such as CT result noted when “CT obtained” marked as “no”) and these charts were re-reviewed. CT results were categorized as well as summarized in free text for review, and the free-text was reviewed for appropriate categorization, with review of the original chart if necessary. Final diagnoses as decided by the treating physician were recorded. Basic demographics were collected on all included patients.

Power calculations were done using the hypothesis that we were likely to find zero dangerous alternative diagnoses, and we would like our 95% CI to stay below 3%. Using the “rule of 3” for rare outcomes, 100 subjects would be needed for our 95% CI to stay below 3%.^23^

### Measurements

The primary outcome was two-fold: dangerous alternative diagnosis as discovered by renal protocol CT and immediate urologic intervention (upon admission). Per our a priori study design, non-renal protocol CTs obtained were not included in the subset of patients with CT, under the presumption that if a CT with contrast (IV or oral) was ordered, renal colic was not the clinician’s primary concern. Secondary outcomes included ED recidivism, admission upon return to the ED, and diagnosis upon return. CT findings were categorized in one of 7 possible categories: 1. Emergent, intervention (surgery/admission/antibiotics) needed immediately (ex. appendicitis, AAA, dissection, diverticulitis, ovarian torsion), 2. Urgent, close follow-up needed (malignancy, unruptured aneurysm), 3. Kidney Stone, likely needing intervention (kidney stone 6mm or larger or severe hydronephrosis), 4. Kidney stone, unlikely needing intervention (5mm or less, no severe hydronephrosis), 5. Non-stone cause of symptoms not needing intervention (ex. Simple ovarian cyst), 6. Incidental findings needing follow-up, 7. Normal. Categories 1 and 2 (Emergent and Urgent) were grouped together as the primary outcome.

### Data Analysis

Data were analyzed descriptively including means or proportions and 95% CIs as appropriate. Stata Version 13 (StataCorp LP, College Station, TX ) was used.

Inter-rater reliability was assessed for 10% of the charts. We choose to check inter-rater reliability on the outcomes that had the most likely chance of being interpreted differently by data abstractors (final ED diagnosis, return to ED in 60 days, note of urologic intervention in 60 days) as well as those that had the most likely chance of affecting the quality of the study (inclusion/exclusion).

## RESULTS

There were a total of 291 patients included after full chart review, with 115 subjects having a non-contrast renal protocol CT (Figure). Demographics are presented in Table 1. At our institution there is only one coding for self-reported ethnicity, rather than one for race and one for ethnicity. Our Hispanic population is primarily Puerto Rican and largely “White – Hispanic” by other coding methods.

### Primary Outcome

Of the 115 encounters that included a renal protocol CT, there were no findings considered emergent or urgent (one-sided 95% CI: [0–2.7%]). Within the 291 subjects presenting with “flank pain” who met inclusion criteria, there were 7 urologic procedures performed upon first admission (2.4%, 95% CI [1.0–4.9%]).

### Secondary Outcomes

Of the 291 subjects, 171 were diagnosed as having renal colic (58.8%) and 11 were admitted, with seven of those undergoing urologic procedures upon admission. Of the 171 diagnosed with renal colic, 51 (29.8%) returned within 60 days for symptoms related to flank pain, and 8 were admitted (4.7%). Of 171 subjects diagnosed with renal colic, there were 29 urologic procedures noted, seven at first admission and 22 at later dates (total 17.0%), however outpatient procedures would be underrepresented in this group due to methodology.

Of the total group, 113/291 (38.8%) returned to the ED within 60 days, and only one was admitted for a non-urological procedure (diagnosis: lap band malfunction), no non-urological surgical emergencies were noted in the returning subjects.

Other imaging included 71 “bedside” or emergency physician-performed renal ultrasounds, and 29 radiology-performed ultrasounds, with 8 patients having both and 20 patients having both a CT and an ultrasound. Bedside ultrasounds were likely under-documented, based on internal reviews of documentation. Of the 92 patients receiving any ultrasound, there were 20 CTs (renal and non-renal protocol) for a proportion of 22%. Of patients not receiving an ultrasound, that proportion was 114 of 199, or 57%. This study was not designed to look at the reasons for this difference. Categorizations and non-stone findings on CT are listed in Table 2. Findings on non-renal protocol CTs are listed in Table 3. No patients in the group who had non-renal protocol CTs had emergent or urgent diagnoses, but it was decided a priori that this group would not be combined with the non-contrast CT group, as the addition of contrast was felt to signify that the provider was looking for something other than an obstructing kidney stone.

Kappas for inter-rater reliability are listed in Table 4. All agreements were found to be “substantial” or “almost perfect.”

## DISCUSSION

This small study suggests that in young patients without urinary tract infection or trauma, the risk of dangerous alternative diagnoses is likely quite low. Since radiation exposure is more concerning in younger patients, further studies regarding the optimum strategy for diagnosing renal colic should consider either stratifying by age or proposing different imaging procedures based on age, such as “ultrasound first.”

In addition to a very low proportion of dangerous alternative diagnoses, only a very small percentage of these young, non-infected patients with renal colic required urgent urologic intervention, suggesting that it may be appropriate to delay CT for non-infected young patients with new-onset renal colic, particularly those who clinically improve in the ED. This is consistent with urology literature suggesting that for non-infected obstructing kidney stones, a trial of Medical Expulsive Therapy is reasonable for stones up to 10mm.^24^

In this study, 7 of the 11 patients initially admitted to the hospital had a urologic intervention during their admission. Since these patients were not admitted because of infection (as that was an exclusion criteria), they were likely admitted due to inadequate pain relief. Perhaps “failure to improve in the ED” warrants further study as one in a set of criterion for a decision rule to help guide CT use.

Lastly, return to the ED for renal colic patients is common and any attempt to decrease CT scanning in this group should keep this in mind. Admission rate upon ED return was low, suggesting that symptomatic control was the driving force for revisit.

## LIMITATIONS

Inherent in any chart review are limitations regarding patient selection and bias, although we set our criteria as rigorously as possible prior to initiating the review and were prudent to avoid the common errors found in chart reviews.^20^–^22^ As stated above, we were not able to fulfill all criteria for methodologic quality in chart reviews.^21^

This chart review was limited to one large hospital. While this hospital has a geographically large and socioeconomically diverse catchment area, it is possible that test utilization at this hospital is slightly different than at other hospitals, which could bias results.

Our study did not seek to evaluate the differences in care received by patients with and without a history of renal colic. We sought to include both groups because the actual management of any individual’s one kidney stone is based more on their clinical presentation at that time than on their history of stone. While it has been argued that those without a history of stones should be subject to a different diagnostic algorithm (for example CT to prove presence of stone), one could argue that need for a test should be determined by its likelihood to change management, not by the desire for simple confirmation.

The majority of patients in our study did not get a CT, so it is possible that surgical emergencies were missed in this group. However, our follow-up data on these patients suggests this is unlikely to be the case; it is likely that these patients did not get CT because physicians noted prior history of multiple CTs or felt they were not concerned about acute pathology other than renal colic.

This study was not designed to look at ultrasound versus CT for the diagnosis of kidney stone. While we collected data on rates of ultrasound, our internal documentation of bedside ultrasound is far from perfect, and ultrasounds reported are an underestimation of unknown magnitude.

This study was done in Massachusetts, which may be a limitation to the generalizability of conclusions about this study, as our patients are overwhelmingly insured by private or public insurance, and therefore generally able to get follow-up with their primary care physician (PCP) or a urologist. In other populations where being seen by a urologist or PCP as an outpatient is not possible, it may be more important to CT these patients while they are in the ED.

Lastly, while our capture of inpatient procedures is likely very accurate (7 procedures for first presentation, out of 291 encounters), our capture of procedures that happened later is not. Not all of our local outpatient surgical centers have operating notes captured by our EMR, and if patients went out of the region, their procedure would not be captured. Similarly, we are unable to say the time period in which these procedures occurred, only that they did not happen on hospital admission. Therefore, if a patient was discharged from the ED but had an outpatient lithotripsy the next day, it may not have been captured. This does leave some gray area for clinicians hoping to have patients avoid immediate CT and instead follow-up with their doctors, as it is unclear how long a patient should wait before seeking either a CT or urologic intervention.

## CONCLUSION

This study adds to the growing evidence that not all patients with suspected renal colic benefit from immediate CT, and provides some evidence that limiting or delaying scanning in non-infected patients under 50 may be safe. Future work should focus on creating algorithms and decision tools to help clinicians avoid immediate CT scanning in these patients.

## Supplementary Information



## Figures and Tables

**Figure:**
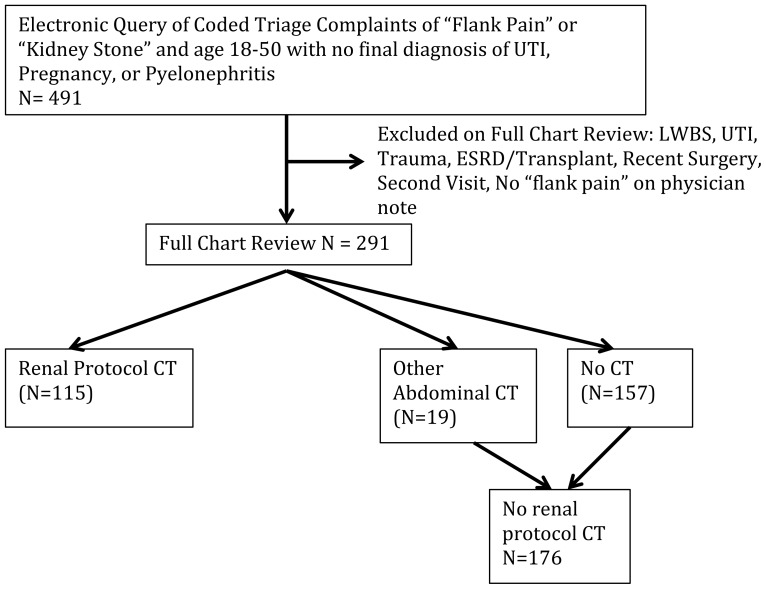
Flow of chart inclusion. *LWBS*, left without being seen; *UTI*, urinary tract infection; *ESRD*, end-stage renal disease; *CT*, computed tomography

**Table 1:** Characteristics of subjects.

Characteristic	All N = 29; N (%)	Renal protocol CT N = 115; N (%)	No renal protocol CT N = 176; N (%)
Mean age (SD)	35.9 (9)	37.0 (9)	35.1 (9)
Sex			
Female	165 (56.7)	63 (54.7)	102 (58.0)
Race			
White	145 (49.8)	56 (48.7)	89 (50.6)
Black	20 (6.9)	9 (7.8)	11 (6.3)
Hispanic	121 (41.6)	49 (42.6)	72 (40.9)
Asian	2 (0.7)	0 (0)	2 (1.2)
Native American	1 (0.3)	0 (0)	1 (0.6)
Not reported	2 (0.7)	1 (0.9)	1 (0.6)
Past medical history			
History of kidney stones	150 (51.5)	50 (43.3)	100 (56.8)
Hypertension	16 (5.5)	6 (5.2)	10 (5.7)
Diabetes mellitus	15 (5.2)	5 (4.3)	10 (5.7)
GERD	9 (3.1)	3 (2.6)	6 (3.4)
Gallstone disease	9 (3.1)	4 (3.5)	5 (2.8)
HIV	8 (2.7)	3 (2.6)	5 (2.8)
Asthma	5 (1.7)	2 (1.7)	3 (2.6)
Medullary sponge kidney	4 (1.4)	0 (0)	4 (2.3)
History of cancer	2 (0.7)	1 (0.9)	1 (0.6)
Final diagnosis of kidney stones by clinician	171 (58.8)	78 (67.8)	93 (52.8)

*CT,* computed tomography; *GERD,* gastroesophageal reflux disease; *HIV,* Human Immunodeficiency Virus

**Table 2:** Renal protocol computed tomography results by category (N =115).

Category	N	Examples
1. & 2. Emergent or urgent	0	
3. Kidney stone > 5mm	7	
4. Kidney stone ≤ 5mm	69	
5. & 6. Possible cause of symptoms, not needing follow-up/intervention and incidental findings needing non-urgent follow-up	14	Cholelithiasis without cholecystitis (3), diverticulosis (2), 18mm adrenal adenoma, primary megaureter, 2.9cm adnexal cystic lesion, 4mm pulmonary nodule, umbilical hernia, 6mm splenule adjacent to spleen, low density liver lesions, hepatic steatosis
7. Negative	25	

**Table 3:** Non-renal protocol computed tomography results by category (N = 19).

Category	N	Examples
1 &2. Emergent or Uurgent	0	
3. Kidney stone > 5mm	3	
4. Kidney stone ≤ 5mm	2	
5. & 6. Possible cause of symptoms, not needing follow-up/intervention and incidental findings needing non-urgent follow-up	9	Ovarian cyst, pulmonary nodule, ovarian mass (later determined benign), possible tuberculosis of bladder, unchanged liver lesions, constipation
7. Negative	5	

**Table 4:** Kappa values calculated for 10% of charts.

Variable	Kappa	95% CI
Inclusion vs. exclusion	0.89	0.73–1.0
Final emergency department diagnosis	0.77	0.52–1.0
Return within 60 days	0.77	0.44–1.0
Urologic intervention within 60 days	1.0	0.67–1.0

## References

[1] Agency for Healthcare Research and Quality Acute onset flank pain — suspicion of stone disease. ACR Appropriateness Criteria.

[2] Fulgham PF, Assimos DG, Pearle MS (2012). American Urological Association (AUA) Guideline.

[3] Westphalen AC, Hsia RY, Maselli JH (2011). Radiological imaging of patients with suspected urinary tract stones: national trends, diagnoses, and predictors. Acad Emerg Med.

[4] Fwu C-W, Eggers PW, Kimmel PL (2013). Emergency department visits, use of imaging, and drugs for urolithiasis have increased in the United States. Kidney Int.

[5] Katz DS, Scheer M, Lumerman JH (2000). Alternative or additional diagnoses on unenhanced helical Computed Tomography for suspected renal colic: experience with 1000 consecutive examinations. Urology.

[6] Hoppe H, Studer R, Kessler TM (2006). Alternate or additional findings to stone disease on unenhanced Computerized Tomography for acute flank pain can impact management. J Urol.

[7] Ahmad NA, Ather MH, Rees J (2003). Incidental diagnosis of diseases on un-enhanced helical Computed Tomography performed for ureteric colic. BMC Urol.

[8] Ha M, MacDonald RD (2004). Impact of CT scan in patients with first episode of suspected nephrolithiasis. J Emerg Med.

[9] Marin JR, Grudzen CR (2014). Emergency physician radiation risk communication: a role for shared decision-making. Acad Emerg Med.

[10] Berrington de Gonzalez A, Mahesh M, Kim K-P (2009). Projected cancer risks from Computed Tomographic scans performed in the United States in 2007. Arch Intern Med.

[11] Mathews JD, Forsythe AV, Brady Z (2013). Cancer risk in 680 000 people exposed to Computed Tomography scans in childhood or adolescence: data linkage study of 11 million Australians. BMJ.

[12] Pearce MS, Salotti JA, Little MP (2012). Radiation exposure from CT scans in childhood and subsequent risk of leukaemia and brain tumours: a retrospective cohort study. Lancet.

[13] Sternberg S Study: Unnecessary CT scans exposing patients to excessive radiation.

[14] Coll DM, Varanelli MJ, Smith RC (2002). Relationship of spontaneous passage of ureteral calculi to stone size and location as revealed by unenhanced helical CT. AJR Am J Roentgenol.

[15] Zwank MD, Ho BM, Gresback D (2014). Does Computed Tomography scan affect diagnosis and management of patients with suspected renal colic?. Am J Emerg Med.

[16] Broder J, Bowen J, Lohr J (2007). Cumulative CT exposures in Emergency Department patients evaluated for suspected renal colic. J Emerg Med.

[17] Moore CL, Bomann S, Daniels B (2014). Derivation and validation of a clinical prediction rule for uncomplicated ureteral stone--the STONE score: retrospective and prospective observational cohort studies. BMJ.

[18] Moore CL, Daniels B, Singh D (2013). Prevalence and clinical importance of alternative causes of symptoms using a renal colic Computed Tomography protocol in patients with flank or back pain and absence of pyuria. Acad Emerg Med.

[19] Smith-Bindman R, Aubin C, Bailitz J (2014). Ultrasonography versus Computed Tomography for Suspected Nephrolithiasis. N Engl J Med.

[20] Vassar M, Holzmann M (2013). The retrospective chart review: important methodological considerations. J Educ Eval Health Prof.

[21] Kaji AH, Schriger D, Green S (2014). Looking Through the Retrospectoscope: Reducing Bias in Emergency Medicine Chart Review Studies. Ann Emerg Med.

[22] Worster AR, Bledsoe D, Cleve P (2005). Reassessing the Methods of Medical Record Review Studies in Emergency Medicine Research. Ann Emerg Med.

[23] Eypasch E, Lefering R, Kum CK (1995). Probability of adverse events that have not yet occurred: a statistical reminder. BMJ.

[24] Preminger GM, Tiselius H-G, Assimos DG (2007). 2007 Guideline for the management of ureteral calculi. Eur Urol.

