# Supplementary material for: Young Patients with Suspected Uncomplicated Renal Colic are Unlikely to Have Dangerous Alternative Diagnoses or Need Emergent Intervention
Source: West J Emerg Med. 2015 Mar 13;16(2):269–75. doi: 10.5811/westjem.2015.1.23272 (PMC4380378; doi:10.5811/westjem.2015.1.23272)
Supplement: Supplementary file 1 [file wjem-16-269-s001.pdf]

Nephrolithiasis

Retrospective Chart Review of Patients with Final Diagnosis of Nephrolithiasis: Can We Safely Decrease CT Scan Use? [Principle Investigator: Schoenfeld, Elizabeth]

DATA COLLECTION FORM

1. Subject Study ID: ______________________
2. MRN:__________________
3. Inclusion: Age >17 [] - Y [] - No (do not continue)
4. Exclusion: Pregnant [] - Y (do not continue) [] - No

5a. Gender: [] - male [] - female

5b. Ethnicity: [] - white, [] - black, [] - asian, []- hispanic, [] - other, 6 - unk

6. Date of visit:
7. History of kidney stones: [] - Yes [] - no
8. History of intervention for KS: [] - Yes [] - no (intervention__________________)
9. Medical History:
    a. none
    b. DM
    c. HTN
    d. CAD
    e. pancreatitis
    f. gallstones
    g. renal disease
    h. AAA
    i. abdominal surgery
    j. Cancer (type ______________)

10. Coumadin [] - yes [] - no
11. Recent trauma [] - yes [] - no

Triage Vital Signs	
12. Temperature (F/C)	
13. Pulse	
14. Systolic BP	
15. Diastolic BP	

Chief Complaint:

16. Location: [] - right, [] - left, [] - both
17. [] - flank, [] - back, [] - abdominal, [] - more than one, (_____)

18a. Duration: ______________hrs

Nephrolithiasis

18b. [] - <6, [] - 6-24, [] - 24-48, [] - 48hrs -1 week, [] - >1 week
19. Physical Exam: Abdominal tenderness noted: []- no, [] – mild, []- moderate, [] – severe; Location: [] RUQ, [] LUQ, [] RLQ, [] LLQ, [] Diffuse, [] Epigastric
19b. Presence of Nausea and vomiting: [ ] – none, [ ] – nausea alone, [ ] – nausea and vomiting

Testing:

20. Udip done [] - y, [] - n
21. Blood []-y, [] - n
22. Leuks []- y, [] -n
23. nitritis [] - y, []- n
24. Formal UA: []- y, [] - n
25. RBCs #________,
26. WBC # ________
27. Cr done [] - Y, [] - N, #____
28. WBC done []- Y, []- N, # ___
29. CT abd done [] - y [] - no
30. CT renal protocol []- y, [] - n
31. CT []- renal, [] - W IV contrast, [] - with po (+/- IV)
32. Bedside Ultrasound [] - y, [] - no
33. CT results: diagnostic of PAIN [] - y, [] - N, [] - unk
34. Findings: [] - no CT, [] - normal, [] - KS ≤5mm, []- KS ≥ 5mm, [] - finding requiring urgent/emergency FU/intervention (___________),[] - finding requiring non-urgent or no FU (________________)
35. Stone size ___________; Location: [] – Prox; [] – Mid; [] UVJ; [] bladder
36. CT Hydronephrosis L: [] - none, [] - mild, [] - moderate, [] - severe, [] - hydro NOS
37. CT Hydronephrosis R: [] - none, [] - mild, [] - moderate, []- severe, [] - hydro NOS
38. Bedside US L: [] - normal, [] - mild hydro, [] - moderate, [] - severe
39. Bedside US R: [] - normal, [] - mild hydro, [] - moderate, [] - severe
40. Formal US L : [] - normal, [] - mild hydro, [] - moderate, [] - severe
41. Formal US R : [] - normal, [] - mild hydro, [] - moderate, [] - severe
42. Final ED diagnosis: [] - kidney stone, [] - other (_____________)
43. Disposition: [] - admitted, [] - discharged
44. If admitted: intervention during this hospitalization: [] - urological, [] - other surgical, [] - none (___________________)
45. Return to ED within 60 days: []- yes, [] – no; related [] –Y, []-N
46. Final Diagnosis second visit: _______________
47. Admitted: [] - yes, [] - no
48. Intervention/procedure: [] - yes, [] - no _______________

Nephrolithiasis

49. Any visits in 60 days requiring urological intervention [] - y, [] - n
50. Stone Score: total ____

Factor	Categories	Points
Sex	Male	2
Timing of pain	<6 hours	3
	6-24	1
	>24	0
Origin (race	Non-black	3
	Black	0
Nausea/vomiting	Both N and V	2
	Nausea alone	1
Erythrocytes	Pos urine dip or rbc >4	3

51. Stones score Categorical [ ] – 0-5 ; [ ] 6-9, [ ] 10-13
